# Congenital Adrenal Hyperplasia due to 11β‐Hydroxylase Deficiency Presented With Leydig Cell Tumor and Testicular Adrenal Rest Tumors: A Case Report

**DOI:** 10.1155/crie/3675251

**Published:** 2026-04-27

**Authors:** Shervin Mossavarali, Faezeh Sehatpour, Shahrzad Mohseni, Mahnaz Pejman Sani

**Affiliations:** ^1^ Endocrinology and Metabolism Research Center, Endocrinology and Metabolism Clinical Sciences Institute, Tehran University of Medical Sciences, Tehran, Iran, tums.ac.ir; ^2^ Department of Endocrinology, Shariati Hospital, School of Medicine, Tehran University of Medical Sciences, Tehran, Iran, tums.ac.ir

**Keywords:** congenital adrenal hyperplasia, infertility, Leydig cell tumor, testicular adrenal rest tumor

## Abstract

Congenital adrenal hyperplasia (CAH) due to 11β‐hydroxylase deficiency is an uncommon disorder characterized by impaired cortisol synthesis, hyperandrogenism, and mineralocorticoid excess. The coexistence of Leydig cell tumors (LCTs) and testicular adrenal rest tumors (TARTs) is rarely reported, highlighting the diagnostic and therapeutic challenges in such cases. A 35‐year‐old man with a history of hypertension and infertility presented with left testicular masses. His medical history was significant for a prior right orchiectomy, with pathology confirming LCT. Subsequent evaluations revealed azoospermia, elevated adrenal androgen levels, adrenocorticotropic hormone (ACTH) and 17‐OH progesterone levels indicative of CAH due to 11β‐hydroxylase deficiency. Imaging studies identified left testicular masses and bilateral adrenal myelolipomas. The patient was managed with oral dexamethasone and eplerenone, resulting in normalization of blood pressure and electrolytes. This case highlights the complexity of CAH presentations with overlapping testicular and adrenal pathologies. Patients with such conditions should be closely monitored and regularly checked for common complications to ensure timely intervention and optimal management.

## 1. Introduction

11β‐Hydroxylase deficiency is an uncommon cause of congenital adrenal hyperplasia (CAH), accounting for a small proportion of classic CAH cases. 11β‐Hydroxylase converts 11‐deoxycorticosterone (DOC) to corticosterone and 11‐deoxycortisol to cortisol, representing the final steps in cortisol synthesis [1]. Elevated levels of DOC can act as a potent mineralocorticoid, causing hypertension and hypokalemia. Impaired cortisol synthesis causes elevated adrenocorticotropic hormone (ACTH) levels and turns steroidogenesis into androgen production, resulting in hyperandrogenemia. This drives virilization of external genitalia in females, puberty, rapid somatic growth, and accelerated bone age in both sexes [2]. Chronic ACTH stimulation is also associated with hyperplasia of ACTH‐sensitive tissues, which can manifest as testicular adrenal rest tumors (TARTs), and prolonged ACTH excess has been associated with adrenal myelolipomas and other adrenal masses [3, 4]. Adrenal myelolipomas are rare benign tumors composed of mature adipose and hematopoietic elements, reported in ~7.4% of patients with CAH, and are thought to result from mesenchymal metaplasia due to ACTH‐driven adrenal stimulation [5, 6].

TARTs are observed in up to 94% of adult patients with CAH, with bilateral involvement occurring in over 80% of cases [7]. Although TARTs are benign, they can disrupt testosterone production and contribute to infertility due to tubular obstruction [8]. Importantly, TARTs can closely mimic Leydig cell tumors (LCTs), the most common stromal testicular neoplasm, both clinically and radiologically. Both lesions may present as painless testicular masses and may be associated with precocious puberty or infertility [3]. However, distinguishing them is crucial because LCTs require surgical excision, while most TARTs improve with optimized glucocorticoid therapy [9]. This report presents a rare case of a 35‐year‐old man who underwent right orchiectomy for LCT at the age of 28, and was later found to have TARTs and bilateral adrenal myelolipomas, ultimately leading to the diagnosis of CAH due to 11β‐hydroxylase deficiency. Written informed consent was obtained from the patient for participation in this study.

## 2. Case Presentation

A 35‐year‐old man was referred to our clinic for evaluation of infertility, testicular masses, and longstanding hypertension. On physical examination, he had a blood pressure of 150/80 mmHg, his short stature was notable, with a final adult height of 152 cm, and areas of skin hyperpigmentation were observed.

The patient’s past medical history began with hypertension, treated with losartan (50 mg/twice a day) since age 20. He reported to be taller than his friends before puberty. Given the size of the mass and concern for a neoplastic lesion, a right orchiectomy was performed, which was subsequently confirmed to be an LCT by immunohistochemistry (Inhibin A: strongly positive). By that time, he had undergone multiple examinations due to infertility. These examinations showed an elevated testosterone level and azoospermia, findings that persisted in subsequent evaluations. His previous semen analysis showed no sperm motility (0% for all motility types), and no sperm count was detected. The seminal fluid volume was 5.0 mL with a pH of 7.6, and the liquefaction time was 20 min. The family history was generally unremarkable.

The patient’s history and clinical presentation raised the suspicion of 11β‐hydroxylase deficiency. Consequently, a related laboratory examination was requested.

After 1 week, during the second visit, he had a blood pressure of 180/90 mmHg. An endocrine evaluation showed an ACTH level of 121 pg/mL (normal range: 7.2–64), an 8 AM cortisol level of 6.7 µg/dL (normal range: 5.1–23), an androstenedione level of >10 nmol/L (normal range: 1.39–6.59), 17‐OH progesterone level of 29 ng/mL (normal range: 0.2–1.97), a luteinizing hormone (LH) of 0.0 IU/L (normal range: 1.7–8.6), a follicle‐stimulating hormone (FSH) of 0.1 IU/L (normal range: 1.5–12.4), a testosterone of 2.7 ng/mL (normal range: 1.64–7.53), low level of plasma renin activity of 0.5 ng/mL/h (normal range: 0.5–4), a serum aldosterone level in upright position of 5.3 ng/dL (normal range: 3.7–43), and low potassium level of 3.2 mEq/L (normal range: 3.5–5). Additionally, persistent hypertension, indicated excessive mineralocorticoid activity. These findings were suggestive of CAH due to 11β‐hydroxylase deficiency.

Tumor marker analysis showed a β‐HCG level of <0.1 mIU/mL (normal up to 2.0), an alpha‐fetoprotein (AFP) of 4.1 ng/mL (normal up to 6.0), and a lactate dehydrogenase of 365 U/L (normal up to 480), further supporting the assessment of his testicular pathology.

Due to the existence of high blood pressure and hypokalemia, he was admitted to the endocrine ward for treatment and further evaluation. With the consultation of urologists, the patient was started on dexamethasone (0.5 mg every night) and eplerenone (25 mg daily) to evaluate the response to the treatment.

**Figure 1:**
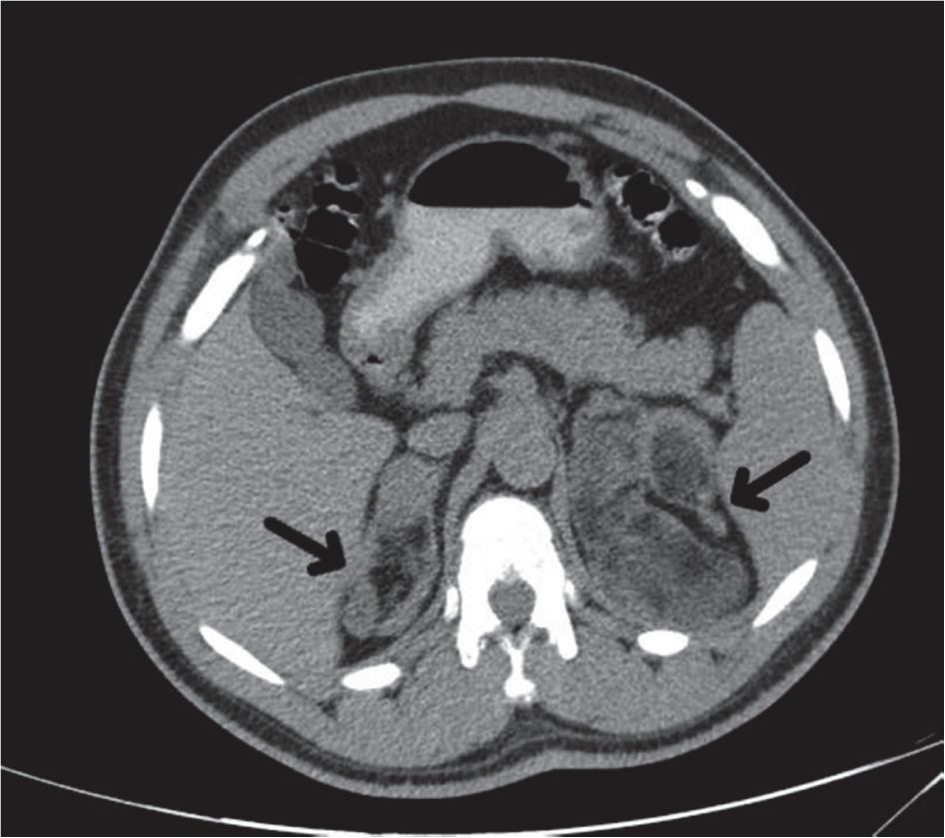
Abdominal CT scan revealing bilateral adrenal myelolipomas: large lesion in the left adrenal (98 mm × 63 mm) and smaller lesion in the right adrenal (49 mm × 24 mm).

On testicular ultrasound during hospitalization, the size of the left testis was 30 mm × 26 mm × 17 mm, and a heterogeneous mass measuring 26 mm × 17 mm with echogenic shadowing and fatty calcifications was reported. Renal and adrenal ultrasound demonstrated two lesions in the right kidney: a round hyperechoic cortical lesion (16 mm × 14 mm) in the upper pole, and a 19 mm × 22 mm lesion at the superior aspect of the upper pole, adjacent to the adrenal gland. Moreover, an ill‐defined hyperechoic cortical lesion (10 mm × 9 mm) was identified in the interpolar region of the left kidney. A subsequent CT scan of the abdomen revealed fat‐containing lesions in both adrenals: a large lesion (98 mm × 63 mm) in the left adrenal and a smaller lesion (49 mm × 24 mm) in the right adrenal, consistent with bilateral adrenal myelolipomas (Figure 1).

Finally, after 10 days, he was discharged in a stable condition with blood pressure of 130/80 mmHg and potassium of 3.8 mEq/L. After a 3‐month follow‐up, the patient’s blood pressure was recorded at 125/80 mmHg. Laboratory examinations revealed potassium levels of 4.1 mEq/L, sodium levels of 139 mEq/L, 17‐hydroxyprogesterone levels of 4 ng/mL, ACTH levels of 60 pg/mL, and plasma renin activity of 20 ng/mL/h. The patient was advised to continue with regular follow‐ups.

## 3. Discussion

TARTs, LCTs, and Leydig cell hyperplasia are the main testicular masses reported in CAH [9]. Typically, in this concept, patients with TARTs are diagnosed when they have a known history of CAH and present with testicular masses [3, 8–10]. However, our patient presented atypically: he had a prior right orchiectomy with a pathological diagnosis of LCT and persistent infertility, and only afterward was CAH identified. Importantly, the patient had not received appropriate endocrine treatment nor regular monitoring for CAH, and this lack of treatment and follow‐up likely contributed to the development of hypertension, progressive TARTs, and adrenal myelolipomas. Due to uncontrolled conditions and the resulting excess 11‐DOC, sodium and water retention occurred, leading to volume expansion and a sustained rise in blood pressure, as observed in this patient. This untreated hormonal imbalance also likely contributed to his short stature, as prolonged excess androgens before puberty can accelerate bone maturation and epiphyseal closure, while inadequate glucocorticoid therapy disrupts normal growth regulation [11].

Differentiating TARTs and LCTs can be challenging at times, as both tumors may have similar clinical findings and consist of polygonal cells that express similar immunohistochemical markers [8]. Clinically, TARTs are more often bilateral and may regress with optimized glucocorticoid therapy because they represent ACTH‐driven adrenal rests, whereas LCTs are typically unilateral, do not respond to steroids, and usually require surgical management [3, 9, 10]. Surgical intervention for TARTs is generally not indicated unless there is chronic intractable testicular pain or progressive mass effect unresponsive to medical therapy [12]. In some cases, TARTs can cause pain, but the primary long‐term concern is loss of fertility from obstructive azoospermia and testicular damage [9]; therefore, semen cryopreservation should be offered as soon as possible once TART is suspected or CAH is diagnosed. Histologically, TARTs often show cord‐like arrangements, extensive fibrosis, and lipochrome pigment, while Reinke crystals are characteristic though not universally present in LCTs [8, 10].

In our patient, bilateral testicular involvement and azoospermia initially suggested TART. However, the painless nature of the masses, elevated serum testosterone, and immunohistochemical findings from the right orchiectomy are consistent with LCT [9]. For the left testis, dexamethasone therapy was initiated, with plans for serial imaging to monitor mass regression and semen analyses to evaluate fertility. If lesions persist or progress, testicular biopsy or surgical intervention may be considered.

Adrenal myelolipomas are benign tumors consisting of myeloid cells and lipomatous [13]. They are typically unilateral and asymptomatic, often discovered incidentally during imaging studies [13]. They are believed to arise from prolonged adrenal stimulation due to elevated ACTH levels in the context of CAH [14]. Notably, patients with inadequately treated or untreated CAH appear to be at a higher risk of developing adrenal masses and lesions [15].

Management decisions are often guided by mass size and its symptoms. Asymptomatic adrenal myelolipomas under 4 cm are usually monitored with periodic CT scans [15]. Masses larger than 10 cm are considered to have an increased risk of hemorrhage and rupture [16]. Laparoscopic adrenalectomy is indicated for lesions that are symptomatic (e.g., pain), suspected to be malignant, and carry out a high risk of bleeding and rupture [16]. In bilateral myelolipomas, the preferred approach is to surgically removing the larger and more symptomatic mass, while preserving the contralateral gland whenever possible. This strategy aims to prevent lifelong steroid dependence [17]. Our case had a medium‐sized myelolipoma in the right adrenal gland and a large one in the left. Since the patient was asymptomatic, we planned for routine follow‐up to monitor the myelolipoma. However, surgical excision may be considered if there is an increase in size or the development of symptoms.

## 4. Conclusion

This complex presentation not only highlights the diverse complications associated with CAH but also points to the challenges in diagnostic evaluation and management strategies, making it a valuable case for reviewing the spectrum of CAH‐related complications and diagnostic and therapeutic approaches.

## Author Contributions

Conceptualization: Mahnaz Pejman Sani. Writing – original draft preparation: Shervin Mossavarali. Writing – review and editing: Mahnaz Pejman Sani, Faezeh Sehatpour, and Shahrazad Mohseni. Data gathering: Faezeh Sehatpour. Supervision: Mahnaz Pejman Sani and Shahrzad Mohseni.

## Funding

This research received no external funding.

## Disclosure

All authors have read and approved the final version of the manuscript. Mahnaz Pejman Sani had full access to all of the data in this study and takes complete responsibility for the integrity of the data and the accuracy of the data analysis.

## Consent

Written informed consent for publication of clinical details and/or clinical images was obtained from the patient.

## Conflicts of Interest

The authors declare no conflicts of interest.

## Data Availability

All data have been presented within the manuscript.
